# GDF15 regulates development and growth of sympathetic neurons to enhance energy expenditure and thermogenesis

**DOI:** 10.1038/s12276-025-01543-9

**Published:** 2025-10-01

**Authors:** Jinyoung Kim, Annie Zhao, Seo Hyun Park, Jaeseok Han, Deok-Hyeon Cheon, Sang-Hyeon Ju, Yoonhyuk Jang, Kon Chu, Hyung Jin Choi, Jiyoon Kim, Myung-Shik Lee

**Affiliations:** 1 Department of Pharmacology, College of Medicine, The Catholic University of Korea, Seoul, Republic of Korea; 2 Department of Medical Sciences, Graduate School, The Catholic University of Korea, Seoul, Republic of Korea; 3 Severance Biomedical Science Institute, Yonsei University College of Medicine, Seoul, Republic of Korea; 4 Soonchunhyang Institute of Medi-bio Science, Soonchunhyang University, Cheonan, Republic of Korea; 5 Department of Anatomy and Cell Biology, Seoul National University College of Medicine, Seoul, Republic of Korea; 6 Graduate School of Medical Science and Engineering, Korea Advanced Institute of Science and Technology, Daejeon, Republic of Korea; 7 Department of Biomedical Sciences, Seoul National University College of Medicine, Seoul, Republic of Korea; 8 Department of Neurology, Seoul National University College of Medicine, Seoul, Republic of Korea; 9 Division of Endocrinology, Department of Internal Medicine, Soonchunhyang University College of Medicine, Cheonan, Republic of Korea

**Keywords:** Neuronal development, Obesity

## Abstract

Growth differentiation factor 15 (GDF15) induces weight loss and increases sympathetic activity through its receptor GFRAL. Given that RET, a GFRAL coreceptor, influences neuronal growth, we studied whether GDF15 can induce the development or growth of sympathetic neurons, in addition to its effect on sympathetic activity. Here we we used *GDF15*-transgenic and *Gdf15*-knockout mice to explore the role of GDF15 in the development and activity of sympathetic neurons. *GDF15*-transgenic mice exhibited increased surface area and volume of sympathetic neurite in adipose tissues. Furthermore, these mice showed heightened energy expenditure, thermogenesis, cold tolerance and an elevated sympathetic response to hypoglycemia. GFRAL was expressed in sympathetic ganglion cells, which was enhanced by GDF15. RET and its downstream signaling molecules such as AKT, ERK and CREB were activated in the sympathetic ganglia by transgenic expression of *GDF15* in vivo or treatment with GDF15 in vitro, an leading to increased expression of genes related to thermogenesis, neurite growth or extension and catecholamine synthesis. An ex vivo treatment of sympathetic ganglia with GDF15 also promoted neurite growth and extension. By contrast, *Gdf15*-knockout mice showed opposite phenotypes, underscoring the physiological role of GDF15 in the development and activity of the sympathetic nervous system. These findings indicate that GDF15 regulates not only the sympathetic activity but also the development or growth of sympathetic neurons through GFRAL expressed in sympathetic ganglion cells, which could contribute to energy expenditure and weight loss. The modulation of GDF15 could be a therapeutic option against diseases or conditions associated with dysregulated sympathetic activity.

## GDF15 boosts sympathetic neuron growth and energy expenditure

Growth differentiation factor 15 is a protein involved in various bodily functions and is linked to diseases such as obesity and cancer. Researchers wanted to understand how GDF15 affects the nervous system, particularly the sympathetic nervous system. This study used genetically modified mice to produce more GDF15, finding that these mice exhibited increased nerve growth in fat tissues, leading to higher energy expenditure and weight loss. Researchers used special imaging techniques to observe nerve growth and measured energy use in mice. They also tested how the mice responded to cold temperatures and insulin, which affects blood sugar levels. This study showed that GDF15 helps nerves grow by activating a specific receptor called GFRAL, which is important for nerve function. The findings suggest that GDF15 could be used to treat conditions related to nerve function and energy use.

This summary was initially drafted using artificial intelligence, then revised and fact-checked by the author.

## Introduction

Growth differentiation factor 15 (GDF15) belongs to the transforming growth factor beta (TGF-β) superfamily and is also known as nonsteroidal anti-inflammatory drug-activated gene 1 (NAG-1), macrophage inhibitory cytokine 1 (MIC-1), placental bone morphogenetic protein (PLAB) or placental TGF-β (PTGFB)^1,2^. GDF15 has been reported to be widely expressed in various tissues, with the highest levels in the liver and placenta^1,2^. Previous studies suggest that GDF15 is induced by various stimuli, including fatty acids and mitochondrial or lysosomal stress^3–5^. Dysregulated GDF15 is implicated in the development of several diseases including cardiovascular diseases, obesity, diabetes or cancer and also in aging or age-related pathology^6^. It has also been reported that serum/plasma GDF15 levels could be a biomarker for diverse diseases such as diabetes, mitochondrial disorders, cardiac failures, renal failures and cancer^6–8^.

Therapeutically, GDF15 is a strong candidate for a treatment option against obesity. Numerous papers reported significant weight loss after GDF15 administration^9,10^. GDF15 is also an important player in cancer cachexia, suggesting a potential role of GDF15 blockade in the management of such a condition^11^. GDF15 acts through GDNF family receptor alpha-like (GFRAL), which has been reported to be restricted in expression to the area postrema (AP) and nucleus tractus solitarius (NTS) of the central nervous system (CNS), leading to anorexia or negative energy balance^12–14^. RET, a GFRAL coreceptor, is a signal transducer activating downstream molecules such as ERK, AKT or PLCγ1^10^. GDF15 has also been reported to enhance sympathetic activity^4,15^, which might be related to weight loss, increased exergy expenditure (EE) or protection against sepsis through fatty acid oxidation or maintenance of hepatic triglyceride export^9,15^. Since RET is involved in the development or growth of neurons and the guidance of axons^16,17^, we hypothesized that GDF15 might induce the development or growth of sympathetic neurons as a neurotrophic factor in addition to its effect on sympathetic activation^4,15^, which might contribute to the GDF15-mediated weight loss through the repair of sympathetic nerve damage and dysfunction imposed by metabolic stress^18^.

## Materials and methods

### Animals

*GDF15*-transgenic (*GDF15*-Tg) and *Gdf15*-knockout (*Gdf15*-KO) mice have been previously described^2,19^. *Gfral*-KO mice were kindly provided by Minho Shong (Korea Advanced Institute of Science and Technology, Daejeon, Korea). C57BL/6 mice were purchased from Orient-Bio Laboratory. Male mice were used in all experiments and housed in an environmentally controlled room (22 °C, 12 h light–dark cycle) with ad libitum access to food and water. All animals were maintained in a specific-pathogen-free facility accredited by the Association for the Assessment and Accreditation of Laboratory Animal Care International. All animal experiments were approved by the Institutional Animal Care and Use Committee of Yonsei University Health System and conducted in accordance with the guidelines of the institutional animal care and use committee.

### Whole mounting and tissue clearing

Whole mounting was conducted essentially on the basis of the immunolabeling-enabled three-dimensional imaging of solvent-cleared organs technique^20^. In brief, after perfusion of anesthetized mice with phosphate-buffered saline (PBS) and then with 4% paraformaldehyde, adipose tissues (epididymal white adipose tissue (eWAT), inguinal white adipose tissue (iWAT) and brown adipose tissue (BAT)) were dissected and fixed in 4% paraformaldehyde at 4 °C overnight. Tissues were washed with PBS for 1 h three times and dehydrated at room temperature in successive 20%, 40%, 60% and 80% methanol for 30 min each, and then twice in 100% methanol for 30 min. Tissues were delipidated at room temperature in 66% dichloromethane in methanol overnight on a rocker and then bleached with 5% H_2_O_2_ in methanol containing 10 mM ethylenediaminetetraacetic acid (pH 8.0) at 4 °C for 48 h. Tissues were rehydrated at room temperature in successive 100%, 80%, 60%, 40% and 20% methanol for 30 min each and were then washed with PBS containing 0.2% Triton X-100 for 1 h twice. Permeabilization was conducted in PBS containing 0.2% Triton X-100, 20% dimethyl sulfoxide (DMSO) and 0.3 M glycine at 37 °C for 24 h, followed by blocking in PBS containing 0.2% Triton X-100, 10% DMSO and 5% donkey serum at 37 °C for 24 h. Tissues were incubated with anti-tyrosine hydroxylase (TH) antibody (Cell Signaling Technology, 58844, 1:500 dilution) in PBS containing 0.2% Tween-20, 10 mg/ml, 5% DMSO and 5% donkey serum at 37 °C for 4 days and were washed in PBS containing 0.2% Tween-20 at 37 °C for 1 h five times. Tissues were then incubated with Alexa 488-conjugated secondary antibody (1:500 dilution) in PBS containing 0.2% Tween-20, 10 mg/ml, 5% DMSO and 5% donkey serum at 37 °C for 3 days and were washed in PBS containing 0.2% Tween-20 at 37°C for 1 h five times. Tissue clearing was conducted by using C Match (Crayon Technologies, 50-3011) as a refractive index solution for 2–3 days. Immunolabeled tissues were pre-embedded in 1% low-melting point agarose prepared in PBS for agar–paraffin double embedding. Tissue blocks were finally cleared with C Match for 2–3 days, and samples were stored at room temperature in the dark until imaging.

### 3D imaging

Optically cleared whole-tissue samples were imaged on a light-sheet fluorescence microscope (Ultramicroscope II, LaVision Biotec) equipped with six fixed light-sheet-generating lenses, a complementary metal–oxide–semiconductor camera (Andor Neo) and a 2×/NA 0.5 objective (MVPLAPO) covered with a 6-mm working-distance dipping cap. Tissue blocks were immersed in a chamber filled with a refractive index solution for the imaging procedure. For imaging at 1.26× effective magnification (0.63× zoom), tissue blocks were scanned by three combined light sheets through each tissue block with a step size of 3 mm. Image stacks were acquired by the continuous light-sheet scanning method using ImspectorPro software (LaVision BioTec). For image analysis, all whole-tissue images were generated using Imaris software (version 9.6.0, Oxford Instruments), and the surface area or volume of TH^+^ neurites were calculated automatically by Filament Tracer tools after 3D reconstruction.

### Metabolic cage

Mice were transported into preautoclaved, sealed transport cages of a PhenoMaster NG system (TSE Systems) and were single housed (22 °C, 12 h light–dark cycle) with ad libitum availability of food and water. Oxygen consumption (VO_2_), carbon dioxide production (VCO_2_), EE, food consumption and locomotor activity were determined in the PhenoMaster NG system for 5 days (2 days of acclimation followed by 3 days of measurement). Locomotor activity was measured by counting the number of infrared beam breaks (XT + YT and Z) during the observation period. XT + YT (combined X- and Y-axis) beam breaks counted horizontal movements across the cage, while Z beam breaks counted vertical movements such as rearing or climbing.

### Cold tolerance test and insulin tolerance test

For the cold tolerance test, mice were transferred from room temperature to 4 °C, and rectal temperature was measured hourly with a TH-5 Thermalert Clinical Monitoring Thermometer (Physitemp) during a 4 °C cold challenge. The insulin tolerance test was conducted in 6-h-fasted mice by an intraperitoneal injection of human insulin (Sigma-Aldrich, I9278) (1 U/kg), and blood glucose levels were measured with an Accu-Check glucometer (Roche). Serum catecholamine (epinephrine and norepinephrine) levels before and 30 min after insulin administration were determined using CatCombi enzyme-linked immunosorbent assay (ELISA) (TECAN, RE59242) according to the manufacturer’s instructions.

### Measurement of BP and HR

Mice were placed on a heat pad at 37 °C and kept for 10 min for acclimation. Blood pressure (BP) and heart rate (HR) were measured using a BP-2000 BP Analysis System (Visitech Systems) and built-in analysis software to acquire and analyze data, before and 1 h after the intraperitoneal injection of human insulin (Sigma-Aldrich, I9278) (1 U/kg). For each measurement, BP and HR were registered 20 times per mouse with a 30-s interval, and the mean value was calculated.

### SCG isolation

The superior cervical ganglia (SCGs) isolated from the lateral side of the carotid artery bifurcation in 8-week-old *GDF15*-Tg, *Gfral*-KO and control mice were collected in Ca^2+^/Mg^2+^-free Hanks’ Balanced Salt Solution (Welgene). To examine the in vitro effect of GDF15, the SCGs from wild-type mice were incubated in 0.25% trypsin for 15 min to remove the epineurium and then treated with 50 ng/ml of recombinant GDF15/MIC-1 (PeproTech, 120–128 C) for 15 min. The SCGs were lysed in a buffer containing protease inhibitors, which was subjected to immunoblot analysis. To estimate the expression of *Gfral* and its downstream genes, total RNA was isolated from the SCGs of 8-week-old *GDF15*-Tg and control mice. The SCGs from *Gfral*-KO and control mice were treated with 50 ng/ml of recombinant GDF15 for 4 h for cDNA synthesis and quantitative reverse transcription polymerase chain reaction (RT–PCR). SCG volume was calculated using a published method estimating ellipsoidal volume: *V* = (length × width^2^)/2 (ref. ^21^).

### SCG explant culture

The SCGs obtained from 8-week-old wild-type and *Gfral*-KO mice were explanted onto poly-ᴅ-lysine-coated culture plates after the removal of epineurium using 0.25% trypsin. Twenty-four hours later, SCGs were treated with 50 ng/ml of recombinant GDF15 in the presence of 1 μM cytosine arabinoside for 3 days to arrest the growth of nonneuronal cells^22^, followed by a treatment with 50 ng/ml of recombinant GDF15 without cytosine arabinoside for 5 days. Optical microscopic imaging was conducted using an Eclipse TS100 microscope (Nikon), and the numbers of neurite outgrowth, branch point and total neurite length were calculated using ImageJ software^23^.

### Quantitative RT–PCR

Total RNA was isolated from tissues (iWAT, BAT and SCG) using TRIzol reagent (Life Technologies) according to the manufacturer’s instructions. cDNA was synthesized using total RNA, M-MLV Reverse Transcriptase (Promega) and oligo(dT) primer. Real-time RT–PCR was performed using a SYBR Green Master Mix (Takara) in a QuantStudio3 Real-Time PCR System (Applied Biosystems). Relative expression values of specific genes for lipid catabolism, thermogenesis, catecholamine synthesis and CREB downstream were normalized to the *Rpl32* mRNA level. Sequences of primers used for real-time RT–PCR are listed in Supplementary Table 1.

### Immunohistochemistry and immunofluorescence

iWAT and BAT were collected and immediately fixed in 10% neutral-buffered formalin (Sigma-Aldrich) to make paraffin-embedded blocks and sections for immunohistochemistry. To investigate the contribution of adrenergic signaling within the tissue in GDF15-induced thermogenesis, iWAT and BAT sections were incubated with antibodies against β3-adrenergic receptor (β3-AR; Abcam, ab94506, 1:150 dilution), hormone-sensitive lipase (HSL; Abcam, ab45422, 1:150 dilution), p-HSL (Ser563)[Ser853] (Affinity Biosciences, AF2350, 1:150 dilution) or UCP1 (Sigma-Aldrich, U6382, 1:100 dilution), followed by incubation with a biotinylated anti-rabbit antibody (Vector Laboratories, BA-1000, 1:100 dilution). After further incubation with avidin–alkaline peroxidase (Vector Laboratories, PK-6100), visualization was achieved using the REAL EnVision Detection System (Agilent Dako, K500711-2) as a chromogenic substrate. Optical microscopic imaging was conducted using an Eclipse TS100 microscope (Nikon).

The whole brains and SCGs were obtained and immediately fixed in 10% neutral-buffered formalin (Sigma-Aldrich, HT501128) to prepare paraffin-embedded blocks. Coronal brain sections, transverse brainstem sections and SCG sections were obtained for subsequent immunofluorescence staining. To detect the expression of the GFRAL receptor in SCGs, brain and SCG sections from wild-type mice were incubated with GFRAL antibody (R&D systems, AF5728, 1:150 dilution) as the primary antibody, followed by incubation with Alexa 594-conjugated anti-sheep IgG (Invitrogen, A11016, 1:200 dilution). Confocal microscopy was performed using LSM780 microscope (Carl Zeiss), and the percentage of GFRAL^+^ cells among total cells as well as the mean fluorescence intensity of GFRAL staining per field area in AP/NTS and SCG sections were quantified using ImageJ (NIH). To investigate GDF15-mediated c-Fos activation and GFRAL induction in sympathetic neurons of the SCGs, SCG sections from *GDF15*-Tg and control mice were incubated with antibodies against TH (Cell Signaling Technology, 58844, 1:150 dilution), GFRAL (R&D systems, AF5728, 1:150 dilution) and c-Fos (Abcam, ab190289, 1:150 dilution) as the primary antibodies. Subsequently, sections were incubated with Alexa 488-conjugated anti-rabbit IgG (Invitrogen, A11008, 1:200 dilution) or Alexa 594-conjugated anti-sheep IgG (Invitrogen, A11016, 1:200 dilution).

To specifically identify sympathetic neurons in the SCGs, confocal microscopy was conducted using LSM780 microscope (Carl Zeiss) after immunofluorescence staining using anti-TH antibody. The percentage of total TH^+^ area and that of TH^+^ area of somas or neurites per total section area were analyzed by using QuPath software (GitHub)^24^. To acquire the TH^+^ immunofluorescence of somas, the boundary of cell bodies was manually drawn. Fluorescence of neurites was calculated by subtracting soma fluorescence from the total neuronal fluorescence.

### Immunoblot analysis

Tissues were lysed in a radioimmunoprecipitation assay buffer containing protease and phosphatase inhibitors. The protein concentration was determined using the Bradford method. Samples (20 μg) were separated on 4–12% NuPAGE Bis-Tris gel (Life Technologies) and transferred to nitrocellulose membranes (Merk Millipore). Membranes were then incubated with antibodies against GFRAL (R&D systems, AF5728, 1:1,000 dilution), p-RET (Tyr1062) (Abcam, ab51103, 1:1,000 dilution), RET (Cell Signaling Technology, 3223, 1:1,000 dilution), p-AKT (Ser473) (Cell Signaling Technology, 9271, 1:1,000 dilution), AKT (Cell Signaling Technology, 9272, 1:1,000 dilution), p-ERK (Thr202/Tyr204) (Cell Signaling Technology, 4370, 1:1,000 dilution), ERK (Cell Signaling Technology, 4695, 1:1,000 dilution), p-CREB (Ser133) (Cell Signaling Technology, 9198, 1:1,000 dilution), CREB (Cell Signaling Technology, 9197, 1:1,000 dilution), TH (Cell Signaling Technology, 58844, 1:1,000 dilution) or β-actin (ACTB) (Santa Cruz Biotechnology, sc-47778, 1:4,000 dilution). After incubating membranes with horseradish peroxidase-conjugated secondary antibodies, immunoreactive protein bands were visualized using EZ-Western Lumi Pico/Femto ECL solution (DoGen). The densitometry of protein bands was performed using ImageJ (NIH).

### Measurement of serum GDF15 levels

GDF15 levels in serum separated from the peripheral blood were determined using Human GDF-15 DuoSet ELISA Kit (R&D Systems, DY957) and Mouse GDF-15 DuoSet ELISA Kits (R&D Systems, DY6385), according to the manufacturer’s instructions.

### Statistical analysis

All values are expressed as mean ± s.e.m. Statistical significance was tested with an unpaired two-tailed Student’s *t*-test to compare two groups and with a two-way analysis of variance with Tukey’s test to compare multiple groups. All analyses were performed using Prism Version 8 software (GraphPad). *P* values less than 0.05 were considered to indicate statistically significant differences.

## Results

### Increased sympathetic innervation in adipose tissue of *GDF15*-Tg mice leads to increased EE

**Fig. 1:**
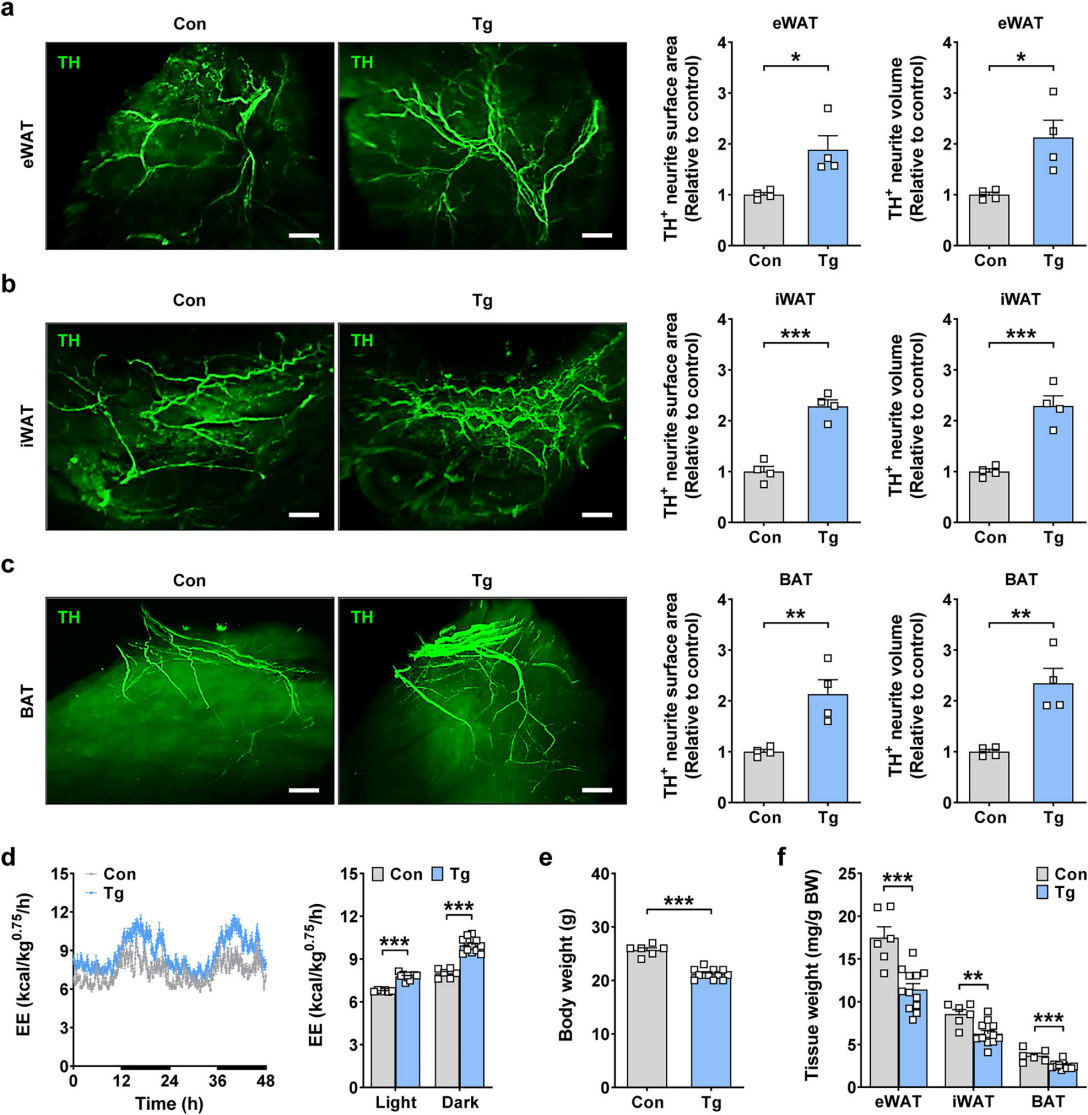
Increased sympathetic innervation in *GDF15*-Tg mice. **a**–**c** Right: the surface area and volume of TH^+^ sympathetic neurites in eWAT (**a**), iWAT (**b**) and BAT (**c**) of *GDF15*-Tg and nontransgenic control mice (*n* = 4 per group). Left: representative fluorescent images of light-sheet microscopy after lipid clearing and whole mounting are shown. Scale bar, 1 mm. **d** The EE of *GDF15*-Tg mice and control mice monitored for 48 h (left) and that in the light or dark cycle (right) (*n* = 6 for Con, *n* = 12 for Tg). The bold lines indicate the dark cycles. **e**, **f** The total body weight (**e**) and fat weight/body weight (**f**) of *GDF15*-Tg mice and control mice (*n* = 6 for Con, *n* = 12 for Tg). All data are shown as means ± s.e.m. Con, control; Tg, transgenic.*P < 0.05, **P < 0.01, ***P < 0.001 by unpaired two-tailed Student’s *t*-test.

First, we investigated sympathetic innervation in eWAT, iWAT and BAT, which are important players in EE, thermogenesis, fatty acid oxidation and lipolysis associated with sympathetic activation^25–27^, in mice overexpressing human *GDF15* (*GDF15*-Tg mice)^19^. Light-sheet fluorescence microscopy (LSFM) after whole mounting and TH immunohistochemistry showed a significantly increased surface area and volume of TH^+^ sympathetic neurites in the eWAT, iWAT and BAT of *GDF15*-Tg mice compared with nontransgenic mice (Fig. 1a–c), suggesting that the transgenic expression of *GDF15* can enhance sympathetic innervation in adipose tissues, which could lead to increased EE, thermogenesis and lipolysis. Consistently, VO_2_, VCO_2_ and EE were significantly elevated in *GDF15*-Tg mice compared with nontransgenic mice during the entire monitoring period, including light and dark cycles (Fig. 1d and Supplementary Fig. 1a,b), indicating the peripheral action of GDF15 in addition to its central action on the CNS. Probably due to increased EE, body weight and fat weight of *GDF15*-Tg mice were significantly lower than those of nontransgenic mice (Fig. 1e, f and Supplementary Fig. 1c, d). Food intake was not significantly different between *GDF15*-Tg and nontransgenic mice (Supplementary Fig. 1e), which could be due to a new equilibrium after the prolonged genetic overexpression of *GDF15*, consistent with previous reports^28^. Locomotor activity was also not different between the two mouse groups (Supplementary Fig. 1f).

### Enhanced thermogenesis and increased sympathetic response of *GDF15*-Tg mice

**Fig. 2:**
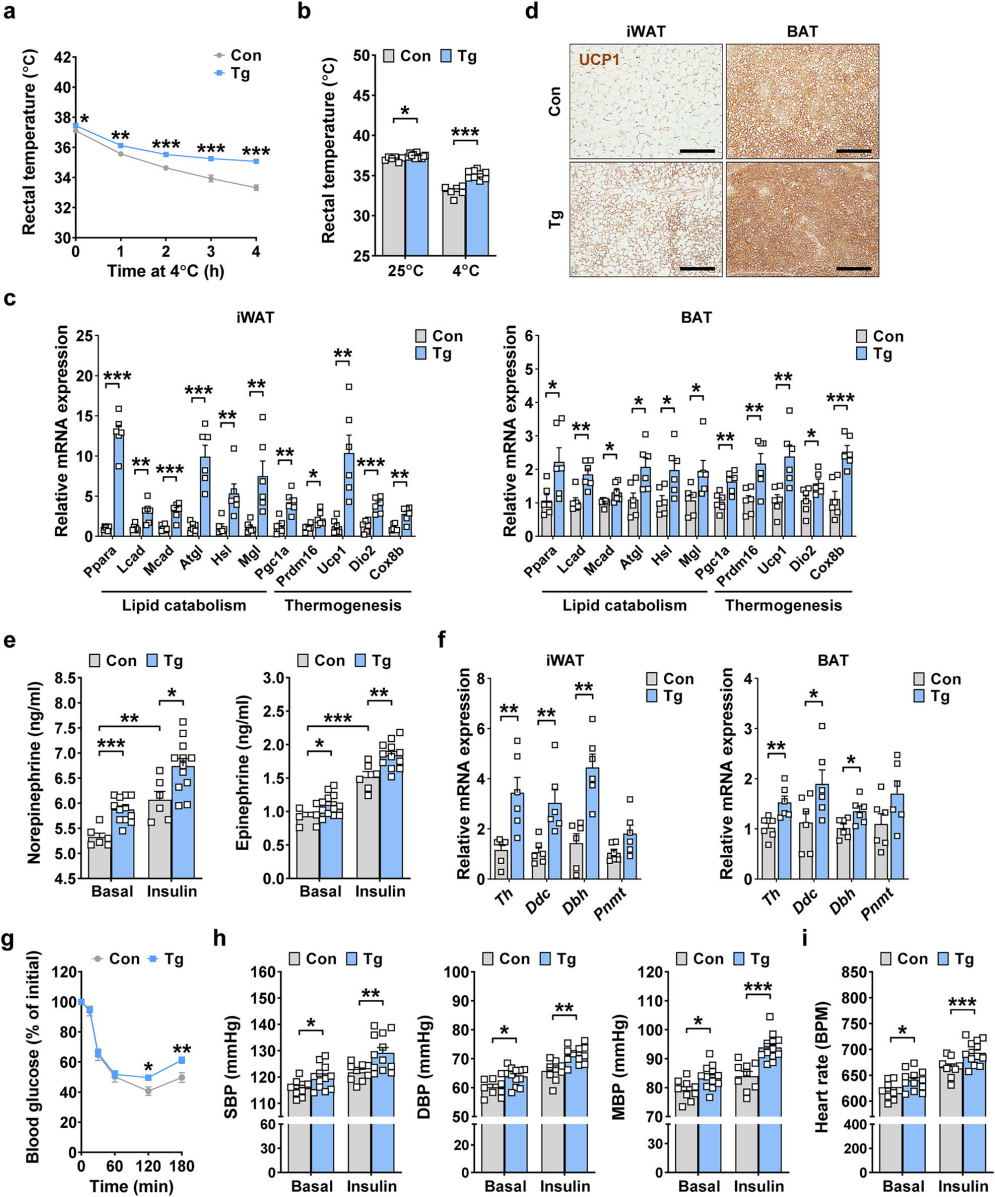
Enhanced sympathetic response of *GDF15*-Tg mice and thermogenesis gene expression in adipose tissue. **a** The rectal temperature of *GDF15*-Tg and control mice monitored at 4 °C for 4 h (*n* = 6 for Con, *n* = 12 for Tg). **b** The rectal temperature of *GDF15-*Tg and control mice at 4 °C and 25 °C (*n* = 6 for Con, *n* = 12 for Tg). **c** The expression of genes of lipid catabolism and thermogenesis in the iWAT and BAT of *GDF15*-Tg and control mice, assessed by real-time RT–PCR (*n* = 6 per group). **d** The UCP1 immunohistochemistry of BAT and iWAT sections from *GDF15*-Tg and control mice. Scale bar, 200 μm. **e** Levels of serum norepinephrine and epinephrine before (basal) and 30 min after insulin injection to *GDF15*-Tg and control mice (*n* = 6 for Con, *n* = 12 for Tg). **f** A real-time RT–PCR using mRNA from the iWAT and BAT of *GDF15*-Tg and control mice and primers specific for the indicated genes of catecholamine synthesis (*n* = 6 per group). **g** Blood glucose levels before (0) and after injection of insulin (1 U/kg) (*n* = 6 for Con, *n* = 12 for Tg). **h**, **i** The systolic BP (SBP), diastolic BP (DBP), mean BP (MBP) (**h**) and heart rate (**i**) of *GDF15*-Tg and control mice before (basal) and 60 min after insulin injection (*n* = 9 for Con, *n* = 12 for Tg). All data are shown as means ± s.e.m. BPM, beats per min. *P < 0.05, **P < 0.01, ***P < 0.001 by unpaired two-tailed Student’s *t*-test.

We next investigated the functional impact of the increased surface area and volume of sympathetic neurites by studying tolerance to cold exposure, which is dependent on thermogenesis and EE^29^. The decline of rectal temperature was significantly less pronounced in *GDF15*-Tg mice compared with nontransgenic mice (Fig. 2a), probably owing to enhanced thermogenesis. Static rectal temperature at room temperature (25 °C) and 4 °C was also significantly higher in *GDF15*-Tg mice compared with nontransgenic mice (Fig. 2b). Consistent with enhanced cold tolerance, the expression of genes related to thermogenesis and lipid catabolism was significantly increased in iWAT and BAT, two important players in thermogenesis and EE, of *GDF15*-Tg mice compared with nontransgenic mice (Fig. 2c). Immunohistochemistry also demonstrated increased expression of UCP1, a critical thermogenic protein, in iWAT and BAT of *GDF15*-Tg mice compared with nontransgenic mice (Fig. 2d). Furthermore, immunohistochemical analyses revealed the increased expression of β3-AR, a major receptor triggering lipolysis and thermogenesis in adipose tissue, along with increased phosphorylation of HSL, a downstream effector of adrenergic signaling, in iWAT and BAT of *GDF15*-Tg mice compared with nontransgenic mice (Supplementary Fig. 2a,b), suggesting that GDF15-mediated thermogenesis is primarily driven by sympathetic neurons and involves a direct action of sympathetic neurons on adipocytes.

We then investigated the hormonal aspect of the sympathetic response by studying the catecholamine response to insulin-induced hypoglycemia^30^. The serum levels of epinephrine and norepinephrine were significantly higher in *GDF15*-Tg mice compared with nontransgenic mice before and 30 min after insulin injection (Fig. 2e), suggesting increased sympathetic activity in both basal and stimulated conditions by the transgenic expression of *GDF15*. Consistent with increased levels of serum catecholamines, expression of catecholamine synthesis-related genes such as *Th*, *Ddc* or *Dbh* was increased in iWAT and BAT of *GDF15*-Tg mice compared with nontransgenic mice (Fig. 2f). Probably due to increased sympathetic response, blood glucose levels 120 and 180 min after insulin injection were significantly higher in *GDF15*-Tg mice compared with nontransgenic mice (Fig. 2g). When we studied systolic, diastolic or mean BP and pulse rate, which are critically regulated by sympathetic tone, they were significantly increased in *GDF15*-Tg mice both before and 60 min after insulin injection compared with nontransgenic mice (Fig. 2h,i), consistent with elevated sympathetic activity by the transgenic expression of *GDF15*.

### Increased expression of GFRAL receptor and activation of GFRAL-expressing neurons in the SCGs of *GDF15*-Tg mice

**Fig. 3:**
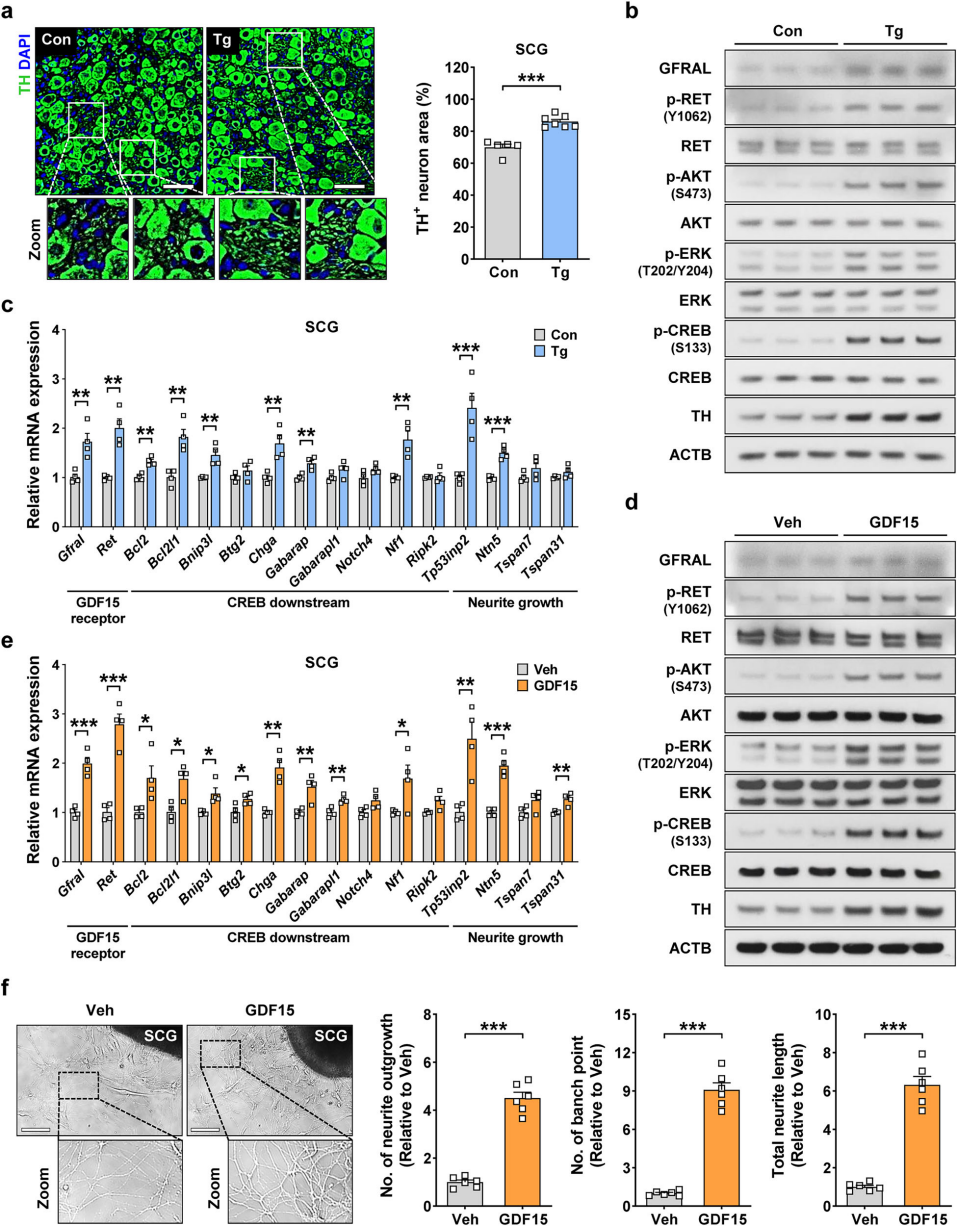
Enhanced GFRAL signaling in the SCGs of *GDF15*-Tg mice and treatment of SCG explant with GDF15 in vitro. **a** Right: the percentage area of entire TH^+^ neuronal cells in SCG sections of *GDF15*-Tg and control mice (*n* = 5 for Con, *n* = 7 for Tg). Left: representative immunofluorescence images are shown. Rectangles are magnified. Scale bar, 50 μm. **b** Immunoblots of tissue extract of the SCGs from *GDF15*-Tg and control mice using indicated antibodies. **c** A real-time RT–PCR using mRNA from the SCGs of *GDF15*-Tg and control mice and primers specific for indicated genes (*n* = 4 per group). **d** Immunoblots of the cultured SCGs treated with recombinant GDF15 or Veh using indicated antibodies. **e** A real-time RT–PCR using mRNA from the cultured SCGs treated with recombinant GDF15 or Veh and primers specific for indicated genes (*n* = 4 per group). **f** Right: the numbers of neurite outgrowth and branch point and total neurite length in the cultured SCGs treated with recombinant GDF15 or Veh (right) (*n* = 6 per group). Left: representative optical images are shown. Rectangles are magnified. Scale bar, 50 μm. All data are shown as means ± s.e.m. Veh, vehicle. *P < 0.05, **P < 0.01, ***P < 0.001 by unpaired two-tailed Student’s *t*-test.

We next investigated the effects of GDF15 on sympathetic neuronal cells using the SCGs that have been widely used for sympathetic nervous system (SNS) research^31^. The volume of the SCGs in *GDF15*-Tg mice was significantly larger than that in control mice (Supplementary Fig. 3a). The TH immunofluorescence demonstrated that the percent area of sympathetic neuronal cells among total ganglion area was significantly increased in *GDF15*-Tg mice compared with control mice (Fig. 3a), suggesting that the sympathetic neuronal mass in the SCGs of *GDF15*-Tg mice would be increased owing to the increases of both total volume of the SCGs and neuronal cell density in the SCGs. When we analyzed the area of somas and that of neurites separately, the percent area of TH^+^ somas was not different between the SCGs of *GDF15*-Tg mice and those of control mice (Supplementary Fig. 3b). By contrast, the percent area of TH^+^ sympathetic neurites was significantly increased in the SCGs of *GDF15*-Tg mice compared with control mice (Supplementary Fig. 3b), suggesting that transgenic *GDF15* expression preferentially expedites the growth of neurites.

**Fig. 4:**
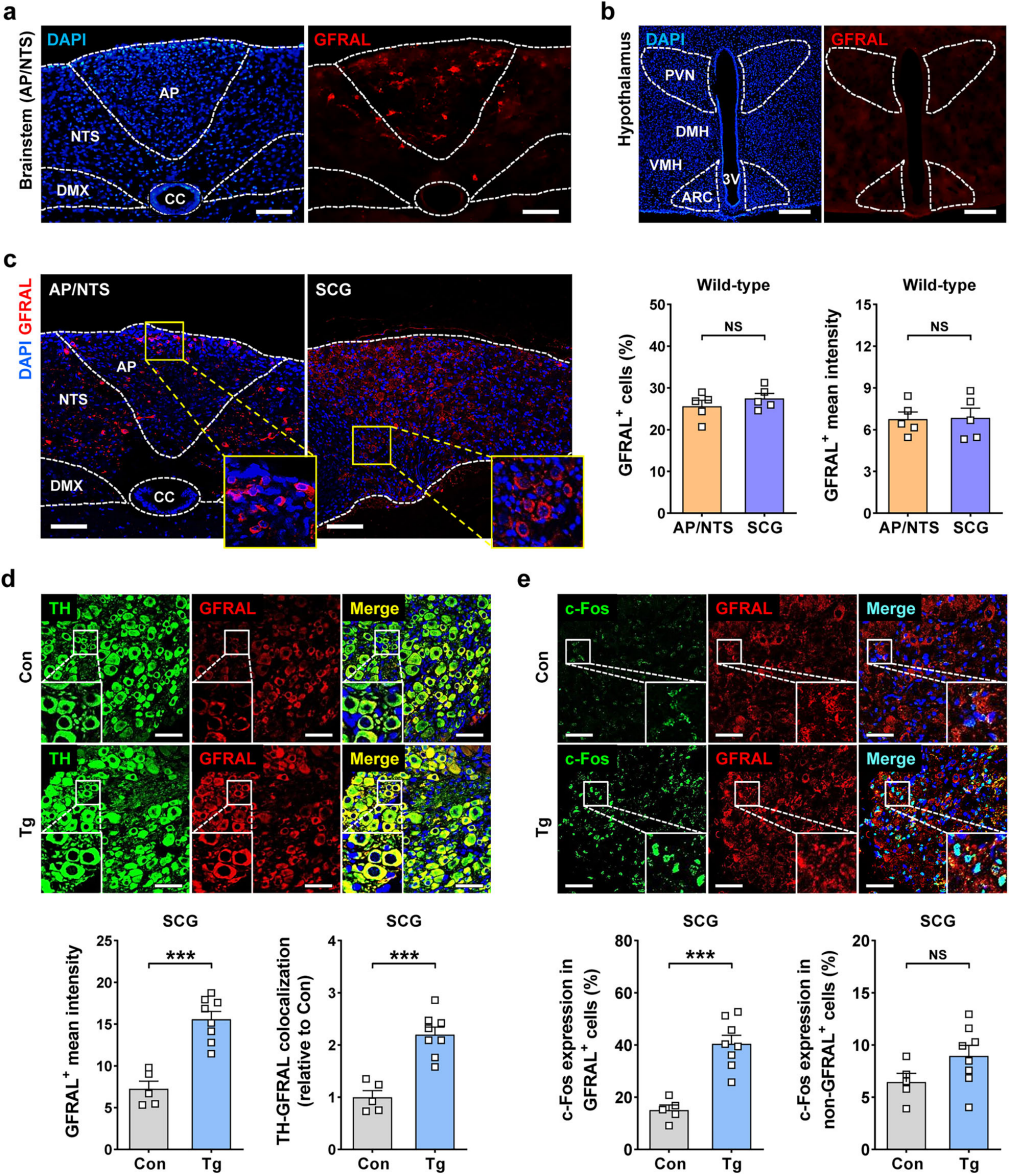
Expression of GFRAL receptor in the SCGs and activation of GFRAL-expressing sympathetic neurons. **a**, **b**, Immunofluorescence images of GFRAL in the brainstem (**a**) and hypothalamus (**b**) of wild-type mice. Scale bar, 100 μm. **c** Right: the percentage of GFRAL^+^ cells and the mean fluorescence intensity of GFRAL staining in AP/NTS and SCG sections of wild-type mice. Left: representative immunofluorescence images are shown. Rectangles are magnified. Scale bar, 100 μm. **d** Bottom: the mean fluorescence intensity of GFRAL staining and colocalization between GFRAL^+^ and TH^+^ neurons in SCG sections from *GDF15*-Tg and control mice (*n* = 5 for Con, *n* = 8 for Tg). Top: representative immunofluorescence images are shown. Insets are magnified. Scale bar, 50 μm. **e** Bottom: the c-Fos expression in GFRAL^+^ and non-GFRAL^+^ cells in SCG sections from *GDF15*-Tg and control mice (*n* = 5 for Con, *n* = 8 for Tg). Top: representative immunofluorescence images are shown. Insets are magnified. Scale bar, 50 μm. All data are shown as means ± s.e.m. DMX, dorsal motor nucleus of the vagus; CC, central canal; DMH, dorsomedial hypothalamus; VMH, ventromedial nucleus of the hypothalamus; 3V, third ventricle; NS, not significant. ***P < 0.001 by unpaired two-tailed Student’s *t*-test.

We next studied the possible expression of GFRAL, the single most important GDF15 receptor, in the SCGs, since GDF15 is more likely to directly induce the growth of sympathetic neurons through binding to the receptor expressed on them rather than to influence their growth through indirect action via the AP/NTS and downstream neurons connecting the brainstem and sympathetic ganglia. Although the roles of neurotransmitters in the synapse formation or guidance of the peripheral nervous system (PNS) has been suggested^32^, the development of the PNS including the SNS is largely controlled by neurotrophic factors such as NGF or GDNF family members^33^. We examined the specificity of the anti-GFRAL antibody using the sections of AP/NTS, well-known sites of GFRAL expression and those of the hypothalamus, a region where GFRAL is reportedly not expressed^14^. Immunofluorescence staining demonstrated the presence of GFRAL-positive cells in the AP/NTS but not in the paraventricular nucleus (PVN) or arcuate nucleus (ARC) of the hypothalamus (Fig. 4a, b), supporting the validity of immunofluorescence staining using an anti-GFRAL antibody. When we studied the possible expression of GFRAL in the PNS using this technique, we observed the clear expression of GFRAL in SCG cells at a similar level to that in the AP/NTS (Fig. 4c), suggesting that GDF15 directly promotes the growth of sympathetic neurons by acting through the receptors expressed on them. When we used the double immunofluorescence method, the GFRAL expression was colocalized with TH fluorescence, showing GFRAL expression on sympathetic neuronal cells (Fig. 4d). The GFRAL expression and colocalization with TH were significantly increased by the transgenic expression of *GDF15* (Fig. 4d). In addition, the expression of c-Fos, a marker of neuronal activity, was significantly elevated in GFRAL^+^ neurons in the SCGs of *GDF15*-Tg mice compared with nontransgenic mice, whereas no significant increase was observed in GFRAL^−^ cells (Fig. 4e), suggesting that GDF15 selectively activates GFRAL^+^ neurons.

### Increased GDF15 downstream signaling through GFRAL-RET in sympathetic ganglion cells

The expression of GFRAL in the SCGs of control mice was further substantiated by real-time RT–PCR and immunoblot analysis, which was also significantly increased by the transgenic expression of *GDF15* (Fig. 3b, c and Supplementary Fig. 4a). When we studied the downstream signal of GFRAL, activation of RET was observed in the SCGs of *GDF15*-Tg mice as shown by increased RET phosphorylation (Fig. 3b), demonstrating the functional effect of GFRAL expression in the SCGs. Downstream of RET, the phosphorylation of AKT, ERK and CREB was observed in the SCGs of *GDF15*-Tg mice (Fig. 3b and Supplementary Fig. 4a). Due to the activation of RET and its downstream signal transducers, the expression of genes related to neurite extension or growth and that of CREB downstream genes related to cell survival such as *Bcl2*, *Bcl2l1* or *Bnip3l* (Gene Set – CREB; maayanlab.cloud)^34^ were elevated in the SCGs of *GDF15*-Tg mice (Fig. 3c). The expression of *Th* and other catecholamine synthesis genes such as *Ddc* or *Dbh* was also increased in the SCGs of *GDF15*-Tg mice (Supplementary Fig. 4b).

We next investigated the effects of GDF15 on the cultured SCGs in vitro to study the cell-intrinsic effects of GDF15 on sympathetic neurons. Similar to the results using intact SCGs, the expression of GFRAL was observed in the cultured SCGs by immunoblot analysis and real-time RT–PCR, which was significantly enhanced by GDF15 treatment in vitro (Fig. 3d, e), strongly supporting the expression of GFRAL on sympathetic neurons and its upregulation by GDF15. GDF15 treatment also significantly activated RET, a GFRAL coreceptor, and AKT, ERK and CREB in RET downstream, as shown by their phosphorylation (Fig. 3d and Supplementary Fig. 4c). Due to the activation of RET and CREB, the expression of genes related to neurite growth or extension and that of CREB downstream genes related to cell survival were increased in the SCGs treated with GDF15 in vitro (Fig. 3e). The expression of *Th*, *Ddc* or *Dbh* related to catecholamine synthesis was also increased in the SCGs treated with GDF15 in vitro (Supplementary Fig. 4d). Consistent with the induction of genes related to neurite growth and extension, the growth and extension of neurites were significantly increased after treatment of the SCGs with GDF15 in vitro as demonstrated by increased numbers of neurite outgrowth, branch point and total neurite length (Fig. 3f).

To directly prove the role of GDF15–GFRAL axis in the sympathetic neurite outgrowth and extension, we used *Gfral*-KO mice and treated their SCGs with GDF15 ex vivo. As hypothesized, neurite growth and extension after treatment with GDF15 was absent in *Gfral*-KO SCGs, as assessed by the number of neurite outgrowths, branch points and total neurite length (Supplementary Fig. 5a). Consistently, the expression of genes related to neurite growth or extension and that of CREB downstream genes were not increased by GDF15 treatment of the SCG of *Gfral*-KO mice (Supplementary Fig. 5b), strongly supporting the role of GDF15 and GFRAL in the growth and extension of sympathetic neurons.

### Reduced sympathetic innervation in *Gdf15*-KO mice

**Fig. 5:**
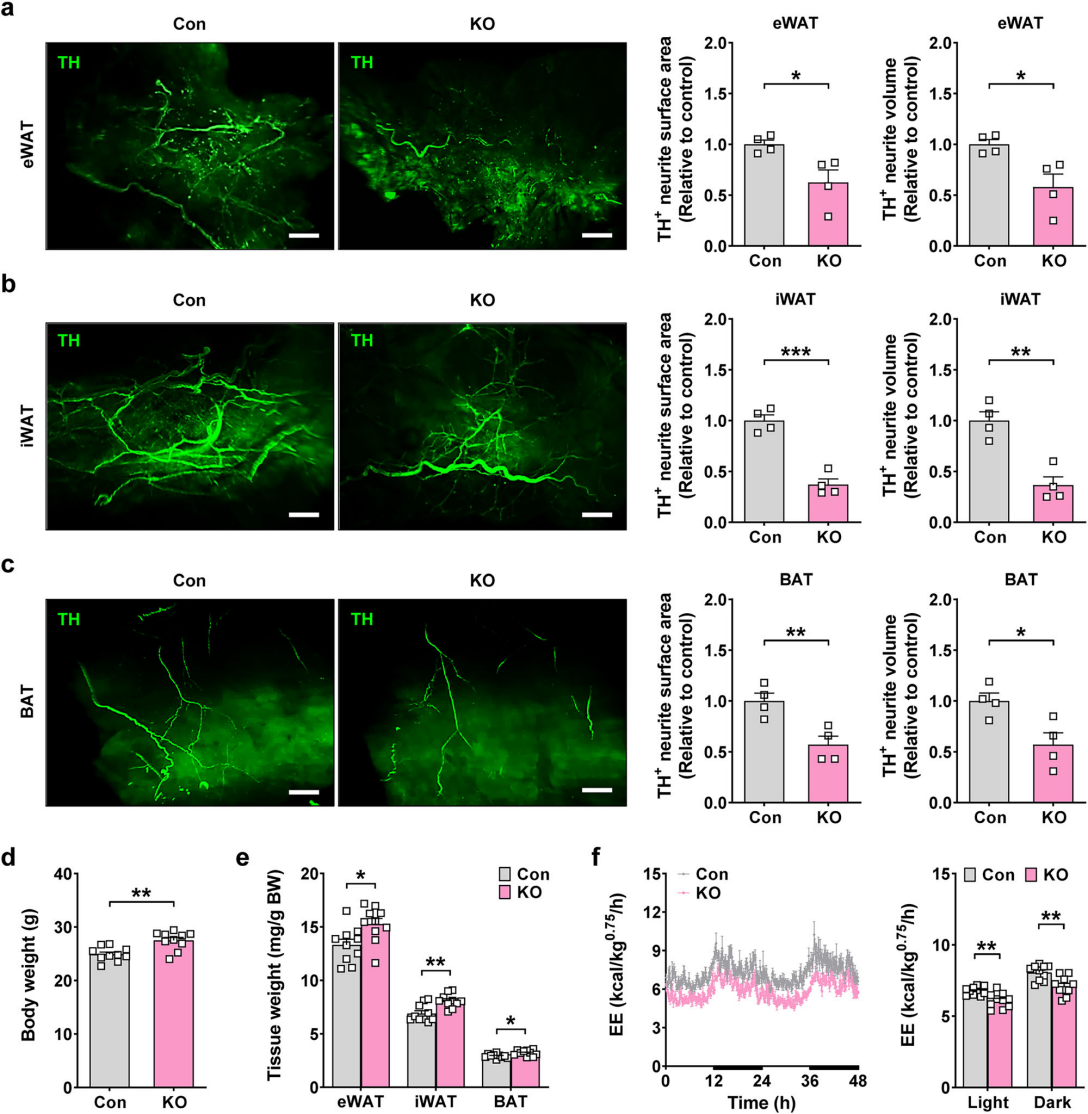
Decreased sympathetic innervation in *Gdf15*-KO mice. **a**–**c** Right: the surface area and volume of TH^+^ sympathetic neurites in eWAT (**a**), iWAT (**b**) and BAT (**c**) of *Gdf15*-KO and control mice (*n* = 4 per group). Left: representative fluorescent images of light-sheet microscopy after lipid clearing and whole mounting are shown. Scale bar, 1 mm. **d**, **e** The total body weight (**d**) and fat weight/body weight (**e**) of *Gdf15*-KO and control mice (*n* = 10 per group). **f** The EE of *Gdf15*-KO and control mice monitored for 48 h (left) and that in the light or dark cycle (right) (*n* = 10 per group). The bold lines indicate the dark cycles. All data are shown as means ± s.e.m. Con, control; KO, knockout; n.s., not significant. *P < 0.05, **P < 0.01, ***P < 0.001 by unpaired two-tailed Student’s *t*-test.

When we determined the human GDF15 level in the serum of *GDF15*-Tg mice by ELISA, it was much higher than murine GDF15 level in the serum of wild-type mice (Supplementary Fig. 6a,b). As this result suggested the possible effect of the supraphysiological level of GDF15 on the SNS, we next studied the sympathetic innervation in adipose tissue of *Gdf15*-KO mice to investigate the physiological role of *Gdf15* related to the sympathetic innervation. In the serum of *Gdf15*-KO mice, GDF15 was not detected, as expected (Supplementary Fig. 6b). In eWAT, iWAT and BAT of *Gdf15*-KO mice, the surface area and volume of TH^+^ sympathetic neurites were significantly lower compared with control mice (Fig. 5a–c), which is inverse to the findings in *GDF15*-Tg mice and suggests a physiological role of endogenous GDF15 in the development of sympathetic neurons.

**Fig. 6:**
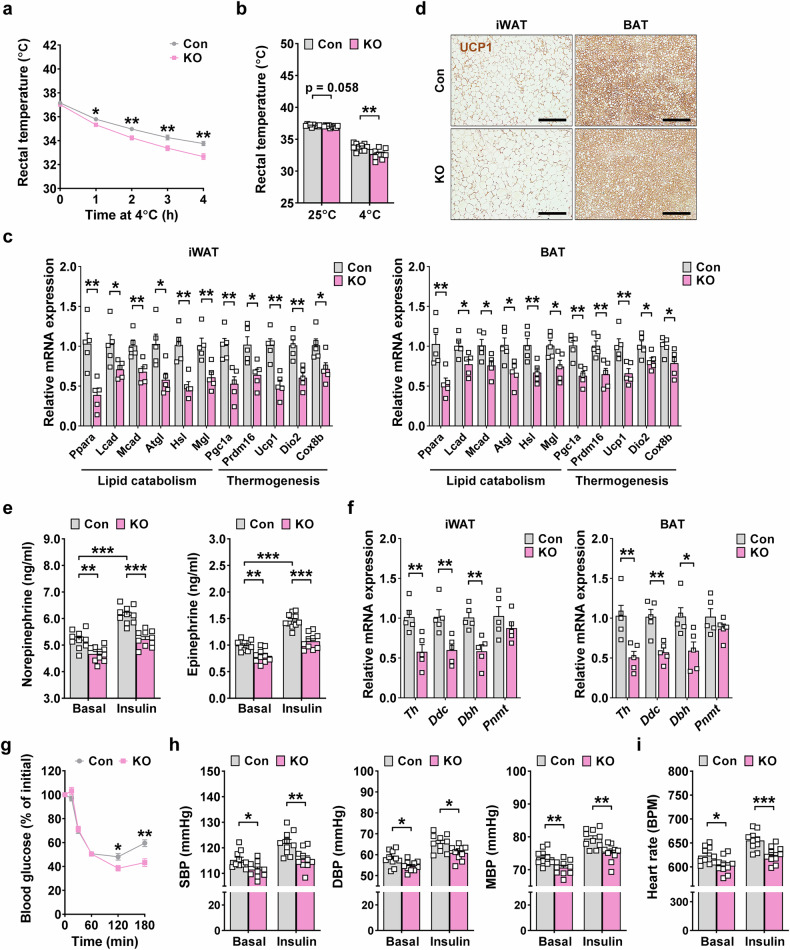
Reduced sympathetic response of *Gdf15*-KO mice and thermogenesis gene expression in adipose tissue. **a** The rectal temperature of *Gdf15*-KO and control mice monitored at 4 °C for 4 h (*n* = 10 per group). **b** The rectal temperature of *Gdf15*-KO and control mice at 4 °C and 25 °C (*n* = 10 per group). **c** The expression of genes of lipid catabolism and thermogenesis in the iWAT and BAT from *Gdf15*-KO and control mice, assessed by real-time RT–PCR (*n* = 5 per group). **d** The UCP1 immunohistochemistry of BAT and iWAT sections from *Gdf15*-KO and control mice. Scale bar, 200 μm. **e** Levels of serum norepinephrine and epinephrine before (basal) and 30 min after insulin (1 U/kg) injection to *Gdf15*-KO and control mice (*n* = 10 per group). **f** The real-time RT–PCR using mRNA from iWAT and BAT of *Gdf15*-KO and control mice and primers specific for indicated genes of catecholamine synthesis (*n* = 5 per group). **g** Blood glucose levels before (0) and after insulin (1 U/kg) injection to *Gdf15*-KO and control mice (*n* = 10 per group). **h**, **i**,The systolic BP (SBP), diastolic BP (DBP), mean BP (MBP) (**h**) and heart rate (**i**) of *Gdf15*-KO and control mice before (basal) and 60 min after insulin injection (*n* = 10 per group). All data are shown as means ± s.e.m. *P < 0.05, **P < 0.01, ***P < 0.001 by unpaired two-tailed Student’s *t*-test.

When we studied the functional role and metabolic effects of endogenous GDF15, total body weight and fat weight were significantly increased in *Gdf15*-KO mice (Fig. 5d,e, and Supplementary Fig. 7a,b), accompanied by decreased VO_2_, VCO_2_ and EE (Fig. 5f and Supplementary Fig. 7c,d), which is inverse to the findings in *GDF15*-Tg mice. Food intake and locomotor activity were not different between *Gdf15*-KO and control mice (Supplementary Fig. 7e,f). Probably due to diminished sympathetic innervation, the tolerance to cold exposure was impaired (Fig. 6a, b). Consistent with impaired cold tolerance, the expression of genes related to thermogenesis and lipid catabolism was significantly downregulated in the iWAT and BAT of *Gdf15*-KO mice compared with control mice (Fig. 6c). Immunohistochemistry also demonstrated a reduced expression of β3-AR and decreased phosphorylation of HSL, as well as diminished expression of UCP1 in iWAT and BAT of *Gdf15*-KO mice (Supplementary Fig. 8a,b and Fig. 6d). Again inverse to the findings in *GDF15*-TG mice, serum levels of epinephrine and norepinephrine were significantly lower in *Gdf15*-KO mice compared with control mice in the basal state (Fig. 6e), suggesting reduced sympathetic activity due to *Gdf15* KO. Consistent with decreased basal levels of serum catecholamines, the expression of catecholamine-synthesis-related genes such as *Th*, *Ddc* or *Dbh* was significantly lower in the iWAT and BAT of *Gfd15*-KO mice compared with control mice (Fig. 6f). After the injection of insulin provoking sympathetic activity, the serum levels of epinephrine and norepinephrine in *Gdf15*-KO mice were significantly lower than those in control mice (Fig. 6e). Probably because of the reduced sympathetic response, blood glucose levels 120 and 180 min after insulin injection in *Gdf15*-KO mice were significantly lower than those in control mice (Fig. 6g), suggesting an impaired response to hypoglycemia. Consistent with the role of endogenous GDF15 in the regulation of sympathetic tone, systolic, diastolic or mean BP and pulse rate were significantly lower in *Gdf15*-KO mice compared with control mice both before and after insulin injection (Fig. 6h,i).

These results suggest that GDF15 plays a role in the development and growth of the SNS neurons contributing to the maintenance of sympathetic tone in addition to its effect on sympathetic neuron excitability. These results also imply a possibility that the modulation of sympathetic activity could be possible using GDF15 or through blockade of GDF15 action, which could have an impact on diverse conditions associated with dysregulated sympathetic activity such as idiopathic orthostatic hypotension characterized by low sympathetic tone^35,36^, hypoglycemic unawareness^37^, hypertension associated with increased sympathetic activity, heart failure^38^, infection, sepsis, shock^39^, obesity^40^ or cancer cachexia^11^.

## Discussion

Previous reports by others and by us have suggested that GDF15 could enhance sympathetic activity^4,15^. Consistently, we observed increased EE, thermogenesis and cold tolerance associated with increased sympathetic activity such as elevated serum catecholamine level in *GDF15*-Tg mice, which could contribute to weight loss by GDF15. By contrast, a recent study reported no effect of GDF15 on rectal temperature, serum catecholamine level or other sympathetic activities in adipose tissues, which could be ascribed to differences in experimental environment, administration dose or schedule leading to dissimilar in vivo concentration and so on^41^. In our additional investigation based on our hypothesis that RET signaling by GDF15–GFRAL interaction might positively regulate the development or growth of sympathetic neurons, we observed increased sympathetic innervation in the adipose tissues of *GDF15*-Tg mice. Our results demonstrating the role of GDF15 in the development of the SNS are reminiscent of the previously reported protective effect of GDF15 on sensorimotor or dopaminergic neurons^42,43^. Although the growth of neurites in the sympathetic ganglia was preferentially increased by the transgenic expression of *GDF15*, that of somas is also likely to be enhanced by *GDF15*. This is because the increase in SCG volume, with a similar percentage of soma area, implies an increased total number or volume of somas in the SCGs of *GDF15*-Tg mice.

Effects of GDF15 on the SNS appear to be mediated by GFRAL, a foremost receptor for GDF15, which was expressed on sympathetic ganglion cells. These results are contrary to previous reports that GFRAL is exclusively expressed in the AP or NTS of the CNS. However, we clearly showed the expression of GFRAL in sympathetic ganglion cells of the PNS, using immunofluorescence, immunoblot analysis and real-time RT–PCR. Furthermore, GFRAL expression in sympathetic ganglion cells was further increased by GDF15 in vivo and ex vivo. Our results demonstrating the effect of GDF15 on cultured SCG cells through GFRAL are in line with a paper reporting that GDF15 had protective effects on retinal ganglion cells through GFRAL expressed on the optic nerves and retina^44^. After GDF15 binding to GFRAL, RET, a coreceptor of GDF15, expressed on ganglion cells, appears to transduce signals for neurite growth or extension and sympathetic function through AKT, ERK or CREB. Effects of GDF15 and GFRAL expressed in the SCGs in the sympathetic neuronal growth was proven by no detectable change of the neurite outgrowth and branch point and total neurite length after ex vivo treatment of the SCGs from *Gfral*-KO mice with GDF15. Together with previous results by others showing the absence of a GDF15-induced activation of c-Fos in sympathetic neuronal cells from mice with targeted disruption of *Gfral*^45^, these results strongly corroborate the role of GDF15 acting on GFRAL in the growth, extension and activation of sympathetic neurons. In the current ex vivo experiment, we used a 50 ng/ml concentration of GDF15. Although the serum level of GDF15 in health subjects is <0.5 ng/ml, serum GDF15 level can increase to ~30–100 ng/ml after therapeutic GDF15 injection or >5 ng/ml in patients with cancer cachexia^41,46,47^. Such results support the clinical or therapeutic relevance of the results from our ex vivo results.

The possibility that GDF15 acts on other potential GDF15 receptors such as CD44 or TGF-βRII^48,49^ cannot be totally eliminated; however, such non-GFRAL effects have been observed mostly in nonneuronal cells, supporting that the GDF15 effects on sympathetic neurons are due to its binding to GFRAL. While our results focused on postganglionic sympathetic neurons, GDF15 might also modulate the development and growth of preganglionic sympathetic neurons through its action on GFRAL in the CNS. Currently known hormones or molecules regulating neuronal development or growth belong to neurotrophin, ciliary neurotrophic factor (CNTF) or the GDNF family^50^. Our data indicate that GDF15 belonging to the GDNF family could also act as a neurotrophic hormone acting on the sympathetic neurons of the PNS, in addition to its effect on the CNS regulating food intake^12–14^.

Our results also suggest a potential of GDF15 modulation as a therapeutic tool in diverse clinical conditions accompanied by abnormal sympathetic activity. In this Article, we did not generate mice with an inducible expression of GDF15 in the adult period, and, thus, we cannot provide genetic evidence proving that GDF15 stimulates growth or maturation of sympathetic neurons when GDF15 is administered in the adult period for therapeutic purposes. However, our ex vivo data showing that GDF15 enhances the growth and extension of neurites, activates RET together with its downstream molecules and induces the expression of catecholamine synthesis genes in sympathetic neurons from adult mice strongly suggest that GDF15 would be able to enhance the growth and maturation of sympathetic neurons in adulthood, which could be related to the effect of GDF15 therapeutically administered to adults after the development of the SNS.

While we focused on sympathetic activity in adipose tissues, GDF15 might affect adrenergic signaling in skeletal muscle as well, which could affect systemic energy expenditure or muscle-specific parameters^41^. Thus, GDF15 might be able to restore impaired sympathetic innervation or activity associated with obesity, high-fat diet, diabetes or aging^18,51–53^, which might help improve the metabolic profile or ameliorate age-related pathology^40,53^. Specifically in the context of obesity, GDF15 administration would be able to promote sympathetic activation and enhance thermogenesis in adipose tissues, aiding in weight management and metabolic regulation. In the case of hypertension, dysregulated sympathetic activity is a well-known contributor to the increased vascular resistance and BP. Modulation of GDF15 by the anti-GDF15 antibody might help restore sympathoadrenal balance and reduce sympathetic overactivity, thereby potentially lowering BP and improving cardiovascular health. By contrast, GDF15 could be a therapeutic candidate against idiopathic orthostatic hypotension characterized by insufficient sympathetic activity. Therefore, pharmacological approaches aimed at modulating GDF15 levels or its signaling pathway could provide new treatment strategies against conditions with dysregulated sympathetic activity including obesity, hypertension or idiopathic orthostatic hypotension.

## Supplementary information

Supplementary Information
