# Supplementary material for: GDF15 regulates development and growth of sympathetic neurons to enhance energy expenditure and thermogenesis
Source: Exp Mol Med. 2025 Oct 1;57(10):2264–76. doi: 10.1038/s12276-025-01543-9 (PMC12586721; doi:10.1038/s12276-025-01543-9)
Supplement: Supplementary file 1 — Supplementary Information [file 12276_2025_1543_MOESM1_ESM.pdf]

Supplementary Fig. 1

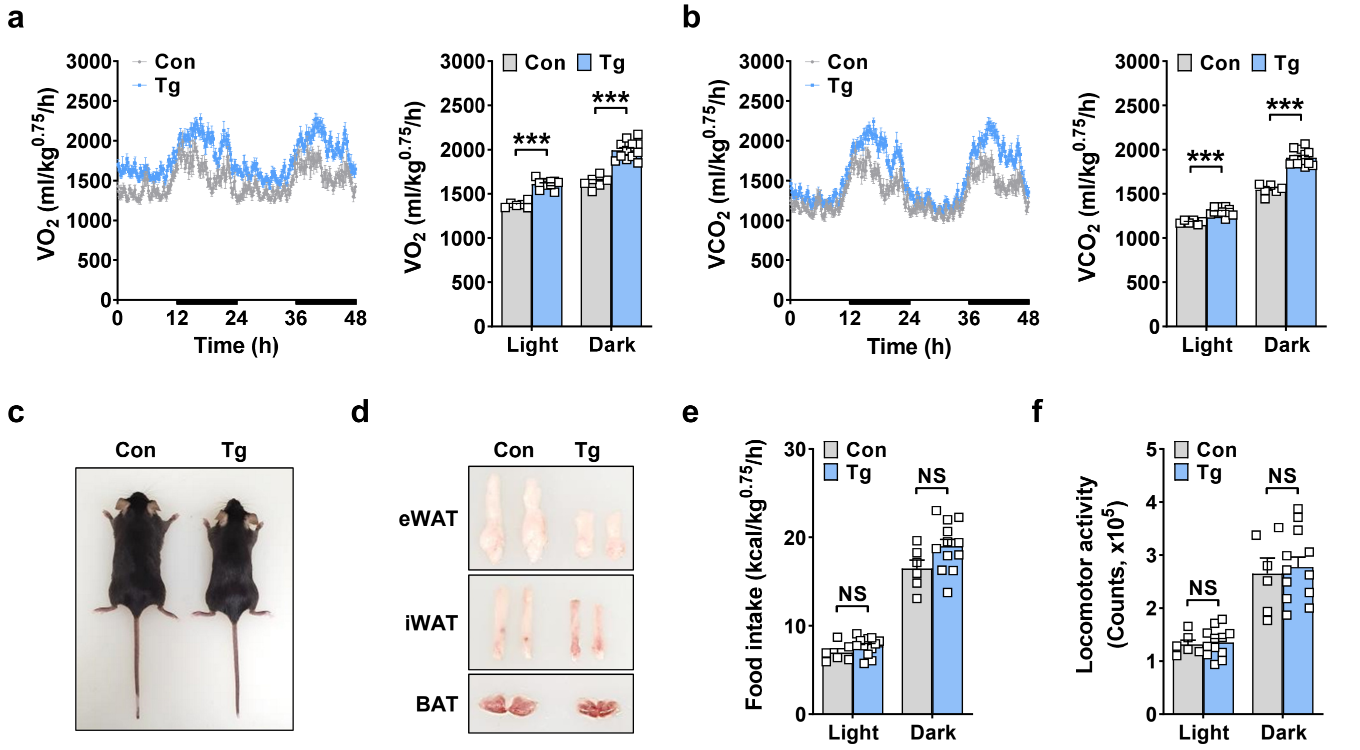

Supplementary Fig. 1│EE and gross images of adipose tissues of *GDF15*-Tg mice. a, **b** VO_2 (**a**) and VCO_2 (**b**) of *GDF15*-Tg mice or non-transgenic mice monitored for 48 h (left), and those in the light or dark cycle (right) (n = 6 for Con, n = 12 for Tg) (Bold lines indicate dark cycles). **c**, **d** Gross images of the whole body (**c**) and eWAT, iWAT or BAT (**d**) of *GDF15*-Tg and non-transgenic control mice. **e**, **f** Food intake (**e**) and locomotor activity (**f**) of *GDF15*-Tg and non-transgenic control mice (n = 6 for Con, n = 12 for Tg). All data are shown as means ± SEM. ***P < 0.001 by two-tailed Student's t-test. (NS, not significant)

Supplementary Fig. 2

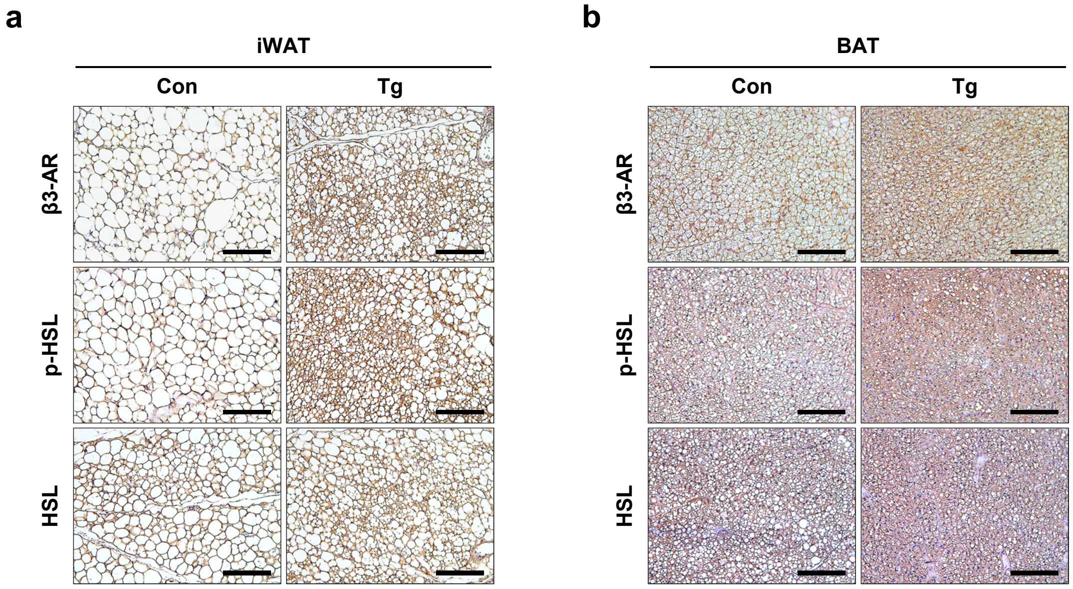

Supplementary Fig. 2│Increased adrenergic signaling and downstream event in adipose tissue of *GDF15*-Tg mice. a, **b** Immunohistochemistry of β3-AR, p-HSL, and HSL in iWAT (**a**) and BAT (**b**) sections from *GDF15*-Tg and control mice. (Scale bar, 200 μm)

Supplementary Fig. 3

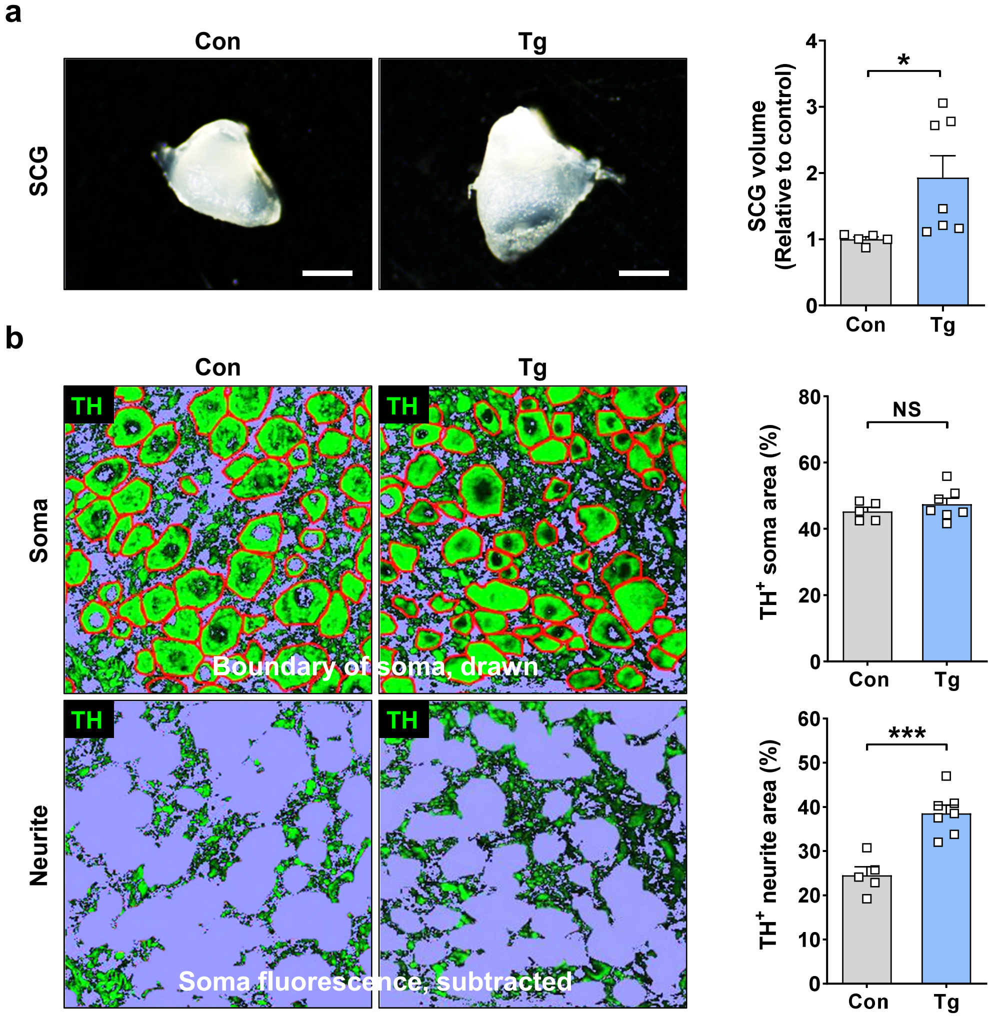

Supplementary Fig. 3│Volume and TH fluorescence of the SCGs. a Quantification of volume of the SCGs from *GDF15*-Tg mice relative to that from control mice determined as described in the Methods (right) (*n* = 5 for Con, *n* = 7 for Tg). Representative gross images are presented (left panel). (Scale bar, 200 μm) **b** Quantification of TH immunofluorescence of somas (right upper) and neurites (right lower) of the SCGs was conducted as described in the Methods (*n* = 5 for Con, *n* = 7 for Tg). Representative fluorescent images of somas (upper row of left panel) and neurites (lower row of left panel) are presented. All data are shown as means ± SEM. *P < 0.05 and ***P < 0.001 by two-tailed Student's *t*-test. (NS, not significant)

Supplementary Fig. 4

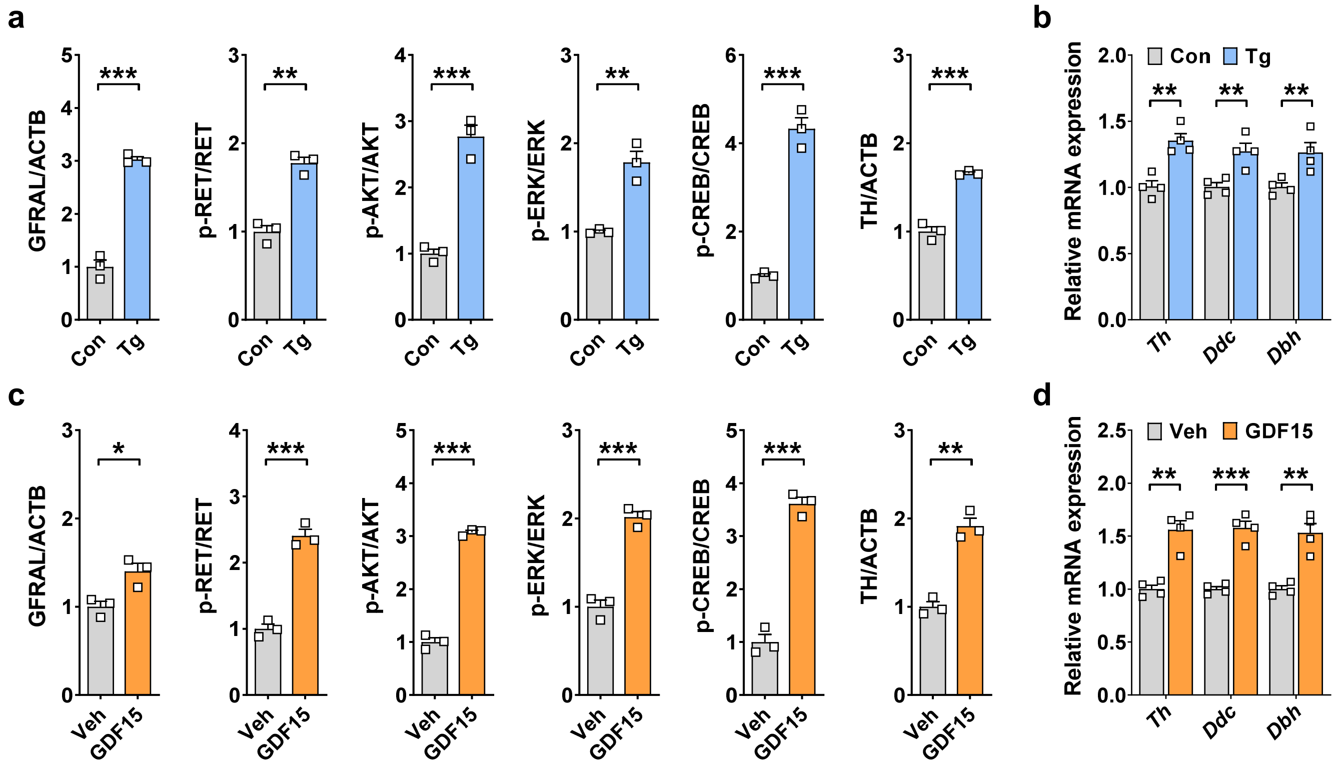

Supplementary Fig. 4│Activation of GFRAL downstream signal in the SCGs. a, c Immunoblot analysis of GFRAL and its downstream signal was conducted using lysate of the SCGs from *GDF15*-Tg and control mice (**a**) or that of the cultured SCGs treated with GDF15 in vitro (**c**). Band intensity was determined by densitometry and normalized to ACTB band intensity (n = 3 per group). **b**, **d** mRNA was isolated from the SCGs of *GDF15*-Tg and control mice (**b**) or from the cultured SCGs treated with GDF15 in vitro (**d**), which was subjected to real-time RT-PCR using and primers specific for indicated genes of catecholamine synthesis (n = 4 per group). All data are shown as means ± SEM. *$P < 0.05$, **$P < 0.01$ and ***$P < 0.001$ by two-tailed Student's t-test.

Supplementary Fig. 5

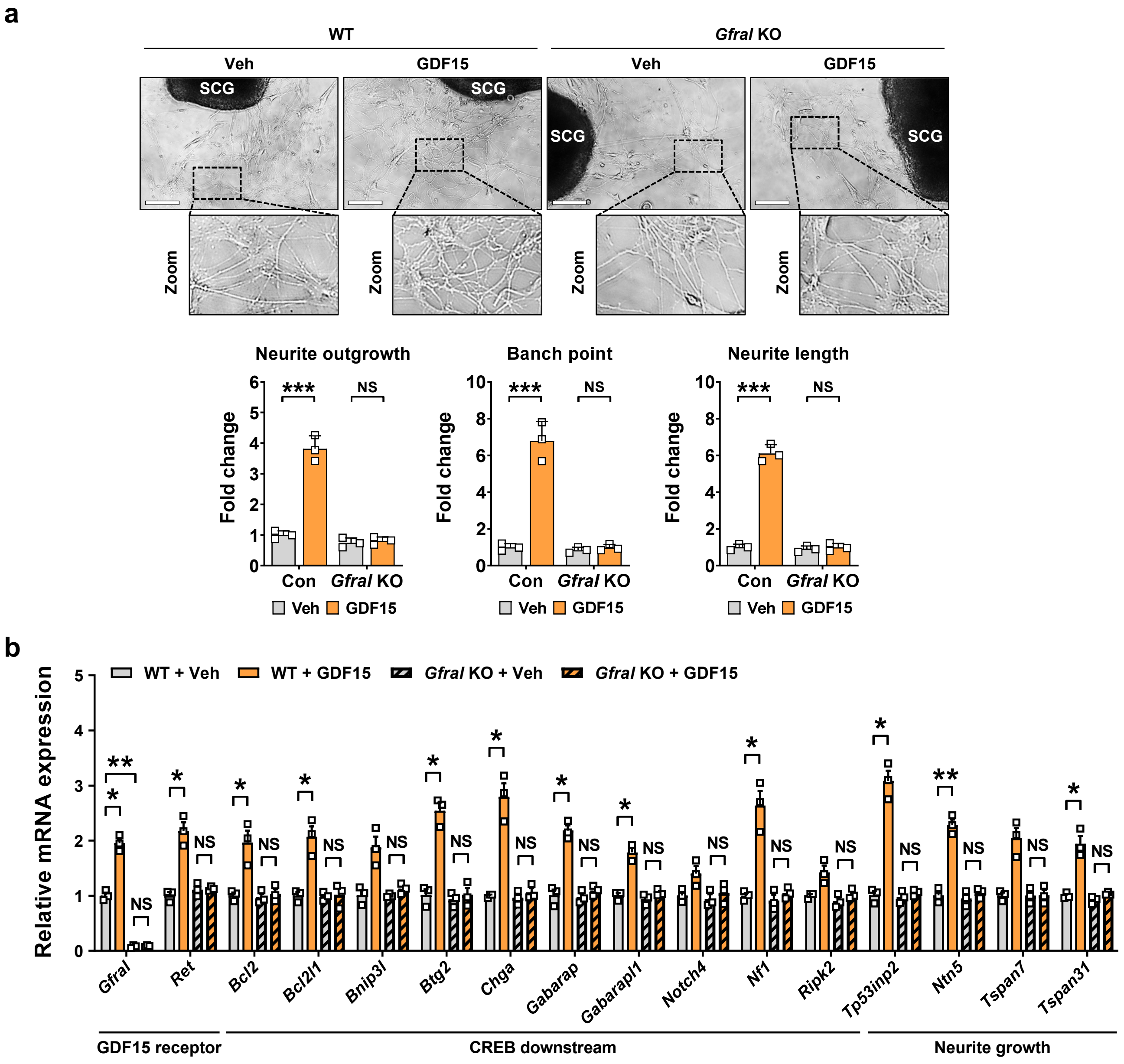

Supplementary Fig. 5│GDF15-induced GFRAL signaling in the SCGs of *Gfral*-KO mice. a The numbers of neurite outgrowth, branch point, and total neurite length in the SCGs from *Gfral*-KO and control mice treated with recombinant GDF15 (lower) (*n* = 3 per group). Representative optical images are shown (upper). Rectangles were magnified. (Scale bar, 50 μm) **b** Real-time RT-PCR employing mRNA from the SCGs from *Gfral*-KO and control mice treated with recombinant GDF15, and primers specific for indicated genes (*n* = 3 per group). All data are shown as means ± SEM. *$P < 0.05$, **$P < 0.01$ and ***$P < 0.001$ by two-way ANOVA with Tukey's test. (NS, not significant)

Supplementary Fig. 6

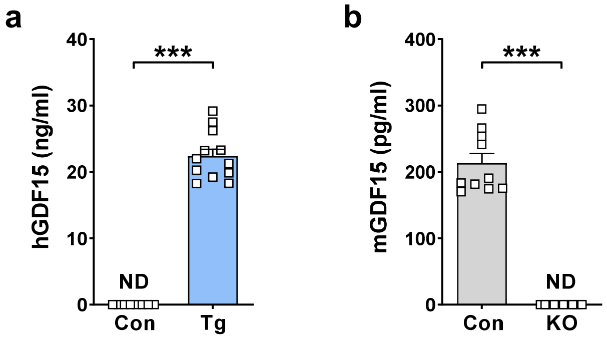

Supplementary Fig. 6│GDF15 levels in the serum of *GDF15*-Tg mice. a Human GDF15 level in the serum of *GDF15*-Tg mice (n = 8 for Con, n = 12 for Tg). **b** Murine GDF15 level in the serum of *Gdf15*-KO mice (n = 10 for Con, n = 10 for KO). All data are shown as means ± SEM. ***P < 0.001 by two-tailed Student's t-test. (ND, not detected)

Supplementary Fig. 7

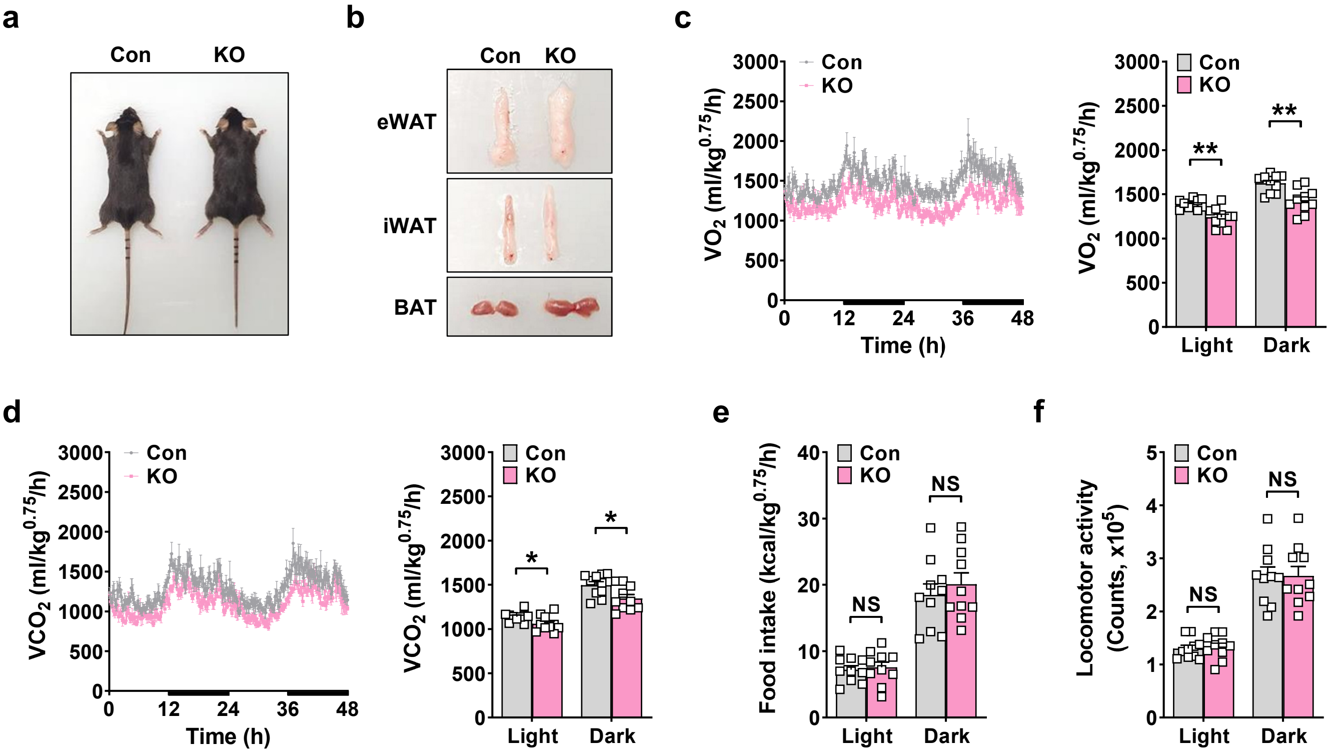

Supplementary Fig. 7│Gross images, EE and food intake of *Gdf15*-KO mice. a, **b** Gross images of the whole body (**a**) and eWAT, iWAT or BAT (**b**) of *Gdf15*-KO and control mice. **c**, **d** VO_2 (**c**) and VCO_2 (**d**) of *Gdf15*-KO mice or control mice monitored for 48 h (left), and those in the light or dark cycle (right) (n = 10 per group) (Bold lines indicate dark cycles). **e**, **f** Food intake (**e**) and locomotor activity (**f**) of *Gdf15*-KO and control mice (n = 10 per group). All data are shown as means ± SEM. *P < 0.05 and **P < 0.01 by two-tailed Student's t-test. (NS, not significant)

Supplementary Fig. 8

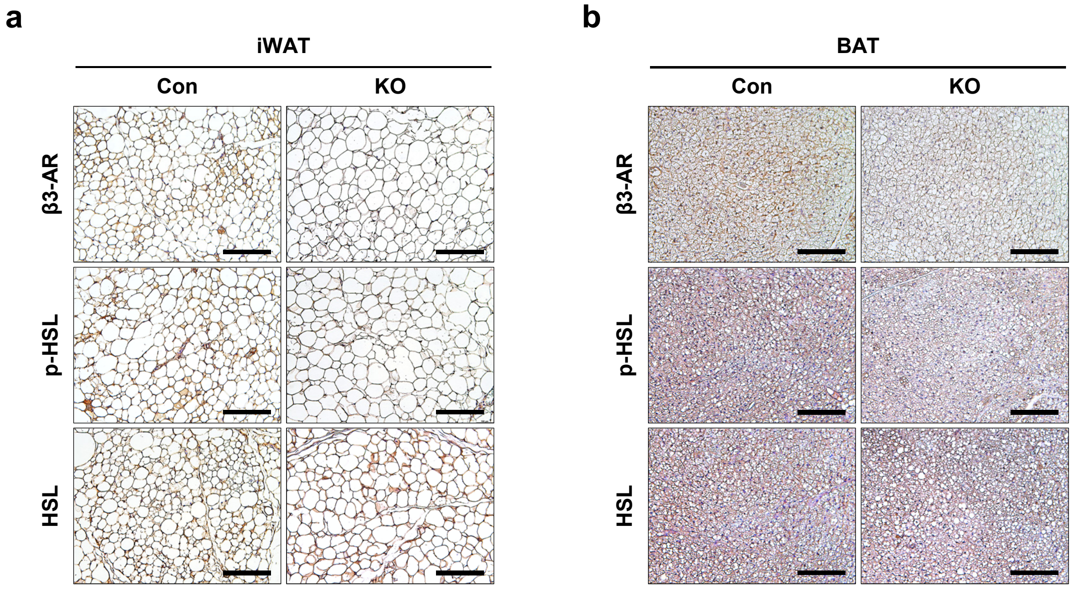

Supplementary Fig. 8 | Decreased adrenergic signaling and downstream event in adipose tissue of *Gdf15*-KO mice. a, **b** Immunohistochemistry of β3-AR, p-HSL, and HSL in iWAT (**a**) and BAT (**b**) sections from *Gdf15*-KO and control mice. (Scale bar, 200 μm)

Supplementary Table 1

Gene	Forward (5’ to 3’)	Reverse (5’ to 3’)
Atgl	CATGATGGTGCCCTATACTC	GTGAGAGGTTGTTTCGTACC
Bcl2	TGACTTCTCTCGTCGCTACC	GGGTGACATCTCCCTGTTGA
Bcl2l1	GTTGGATGGCCACCTATCTG	CACTTCCGACTGAAGAGTGAG
Bnip3l	TCTCACTTAGTCGAGCCGC	GACTCATGCTGTGCATCCAG
Btg2	GAAGAGAACCGACATGCTCC	AGCCAGAACCTTTGGATGGT
Chga	CCAAGGTGATGAAGTGCGTC	CTTGGAGAGCCAGGTCTTGA
Cox8b	GAACCATGAAGCCAACGACT	GCGAAGTTCACAGTGGTTCC
Dbh	TGGAGCTCGGACTGGTGTAT	GCACTTGTCTGTGCAGTAGC
Ddc	CGGCTAAAGGGCTCCAATGA	CGGAGACGACATGGAACCAA
Dio2	GGGACTCCTCTCTGTCTTTT	CCAACTTCGGACTTCTTGTA
Gabarap	CAAAGAGGAGCATCCGTTCG	GCACGGAGATGAATTCGCTT
Gabarapl1	AGGACCACCCCTTCGAGTA	TAAGGCGTCCTCAGGTCTCA
Gfral	CCACATAACTGGGAAGTGCC	GCTGGAAAATCATGCACACG
Hsl	AAGGACTTGAGCAACTCAGA	TTGACTATGGGTGACGTGTA
Lcad	GGGAATGAAAGCTCAGGACA	AGAATCCGCATTAGCTGCAT
Mcad	AGGTTTCAAGATCGCAATGG	CTCCTTGGTGCTCCACTAGC
Mgl	GACGGACAGTACCTCTTTTG	AGAAAAGTAGGTTGGCCTCT
Nf1	CAAAGGATTCCCTCCTCGCA	CACTTTGGGAAACACAACGC
Notch4	GTCTTAAGGCACTGAAGCCAG	TCCAGAACCTCCGATTCACA
Ntn5	GGATTATGTTCTGCACGCCC	CTGTTTGAACACAGCCAGCA
Pgc1a	GCAGCCAAGACTCTGTATGG	CGCTACACCACTTCAATCCA
Pnmt	CGGGACGGGTTCTCATTGAT	CAGCTCCTGACGGTTGACTT
Ppara	GGATGTCACACAATGCAATTCG	TCACAGAACGGCTTCCTCAGGT
Prdm16	CAGCACGGTGAAGCCATTC	GCGTGCATCCGCTTGTG
Ret	GGCTGCATGAGAATGACTGG	TGGGAAGGTGTCGTTGATGA
Ripk2	CCTTAACCAATCGCTGGACG	AGGGAAATTTGAAGGCGGTG
Rpl32 (L32)	CAGTCAGACCGATATGTGAA	TAGAGGACACATTGTGAGCA
Th	GTTTGACCCGTACACCCTGG	CTCTAAGGAGCGCCGGATG
Tp53inp2	CATCGACCTACAGGACAGCTA	GACATGCTGGGATGCTCAATG
Tspan7	CATCGAGGAGAATGGAGACCA	ACGGCATGTAGCAAAGCATC
Tspan31	ATGGTTTGCGGCGGATTTG	GAATGCTAGACACCACACCG
Ucp1	TACTGTCAGCTCTTGTTGCC	TGTACATGGACATCGCACAG

Supplementary Table 1│List of primer sequences for real-time RT-PCR. Relative expression values of specific genes for lipid catabolism, thermogenesis, catecholamine synthesis, and CREB downstream were assessed using mouse primer sequences listed.
